# Direct reprogramming of fibroblasts into skeletal muscle progenitor cells by transcription factors enriched in undifferentiated subpopulation of satellite cells

**DOI:** 10.1038/s41598-017-08232-2

**Published:** 2017-08-14

**Authors:** Naoki Ito, Isao Kii, Noriaki Shimizu, Hirotoshi Tanaka, Shin’ichi Takeda

**Affiliations:** 1 Department of Molecular Therapy, National Institute of Neuroscience, National Center of Neurology and Psychiatry, Tokyo, 187-8502 Japan; 2 Department of Rheumatology and Allergy, IMSUT Hospital, The Institute of Medical Science, The University of Tokyo, Tokyo, 108-8639 Japan; 3 Pathophysiological and Health Science Team, Imaging Application Group, Division of Bio-Function Dynamics Imaging, Riken Center for Life Science Technologies, Hyogo, 650-0047 Japan; 4 Division of Rheumatology, Center for Antibody and Vaccine Therapy, IMSUT Hospital, The Institute of Medical Science, The University of Tokyo, Tokyo, 108-8639 Japan

**Keywords:** Muscle stem cells, Reprogramming

## Abstract

Satellite cells comprise a functionally heterogeneous population of stem cells in skeletal muscle. Separation of an undifferentiated subpopulation and elucidation of its molecular background are necessary to identify the reprogramming factors to induce skeletal muscle progenitor cells. In this study, we found that intracellular esterase activity distinguishes a subpopulation of cultured satellite cells with high stemness using esterase-sensitive cell staining reagent, calcein-AM. Gene expression analysis of this subpopulation revealed that defined combinations of transcription factors (*Pax3*, *Mef2b*, and *Pitx1* or *Pax7*, *Mef2b*, and *Pitx1* in embryonic fibroblasts, and *Pax7*, *Mef2b* and *MyoD* in adult fibroblasts) reprogrammed fibroblasts into skeletal muscle progenitor cells. These reprogrammed cells formed Dystrophin-positive mature muscle fibers when transplanted into a mouse model of Duchenne muscular dystrophy. These results highlight the new marker for heterogenous population of cultured satellite cells, potential therapeutic approaches and cell sources for degenerative muscle diseases.

## Introduction

Satellite cells (SCs) are stem cells in adult skeletal muscle that originate from Pax3/Pax7-expressing muscle progenitors in the dermomyotome^1,2^. SCs are characterized by expression of Pax7 and are mitotically quiescent under physiological conditions. In response to several stimuli, SCs are activated, proliferate, undergo self-renewal, and differentiate into mature muscle fibers^1,3^. Proliferating SCs express MyoD and/or Myf5, which play key roles in determining muscle cell fate, and undergo myogenic differentiation through upregulation of several transcription factors (TFs) including Myogenin and Mrf4.

MyoD is the first identified reprogramming factor that transdifferentiates fibroblasts into skeletal muscle cells^4^. Exogenous expression of MyoD, however, exerts anti-proliferation effects^5^ and therefore cannot maintain the cell state into the proliferating skeletal muscle progenitor cells. Although exogenous expression of MyoD induces differentiated skeletal myocytes, they are not a suitable cell source for several therapeutic applications, including transplantation therapy. Induction of skeletal muscle cells from embryonic stem cells (ESCs) or induced pluripotent stem cells (iPSCs) by exogenous expression of Pax3 or Pax7^6,7^, or from mesenchymal stem cells by exogenous expression of PAX3^8^ have been reported. However, direct reprogramming of non-muscle cells into induced skeletal muscle (iSkM) progenitor cells, which do not pass through a pluripotent stem cell state, have not been reported. Because differentiation-related TFs, such as Myogenin or Mrf4, might inhibit the induction of skeletal muscle progenitor cells, separation of an undifferentiated subpopulation with high stemness from the heterogeneous SC population^1,9–17^ is necessary to identify TFs essential for inducing iSkM progenitor cells.

## Results

In the previous study, we analyzed intracellular Ca^2+^ levels ([Ca^2+^]_i_) using fluorescent Ca^2+^ indicator, Fluo-4 AM^18^. Interestingly, we observed stronger Fluo-4 fluorescence in differentiated C2C12 myotubes compared with that in undifferentiated cells even before stimulation with Ca^2+^ ionophores or agonists (data not shown). We first hypothesized that this basal differences in the fluorescence intensity of Fluo-4 were due to the differences in [Ca^2+^]_i_ between differentiated and undifferentiated cells. However, unexpectedly, we also observed the similar differences in the fluorescence intensity using [Ca^2+^]_i_-independent cell-staining reagents, such as Calcein-AM, fluorescein diacetate (FDA), BCECF-AM and carboxyfluorescein succinimidyl ester (CFSE) (Supplementary Fig. 1). These results suggested that the differences in the fluorescence intensity were originated from the common property of used fluorescent reagents. These reagents have a common chemical structure of acetoxymethyl ester (AM). AM is used for wide variety of reagents to enhance the membrane permeability^19^. AM-based cell-staining reagents are digested by unspecific esterases after which cells are labeled with fluorescence^19^, suggesting that differences in fluorescence intensity were derived from the differences in the esterase activity between differentiated and undifferentiated cells.

**Figure 1:**
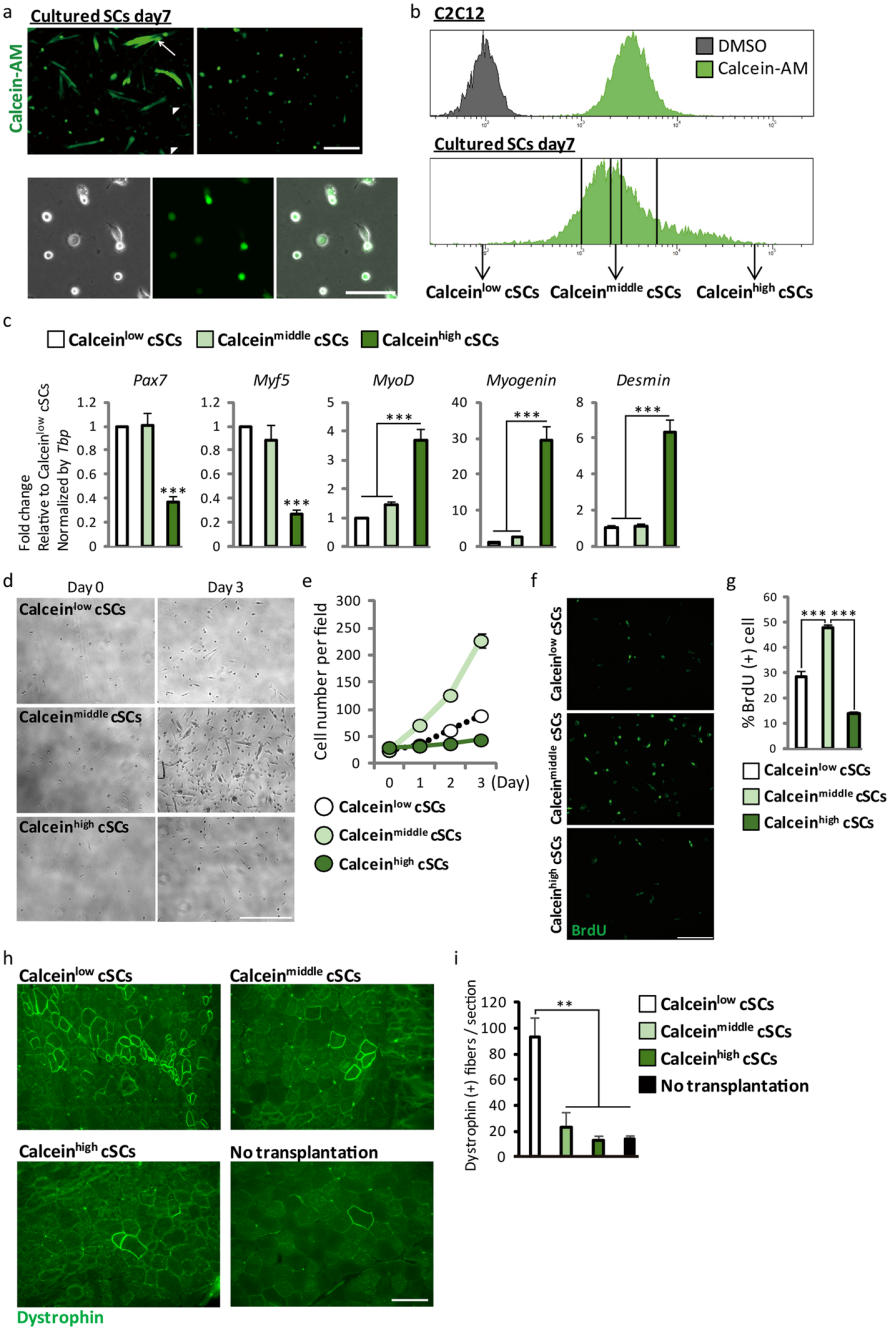
Separation of functionally distinct subpopulation of primary cultured satellite cells by Calcein-AM. (**a**) Representative fluorescence images of calcein-AM-treated cSCs 7 days after isolation. Upper left images show strong fluorescence in differentiated myotubes. Arrow and arrowhead show differentiated and undifferentiated cells, respectively. Upper right and lower images show heterogeneous fluorescence in round, undifferentiated cells in low-power and high-power fields, respectively. *n* > 3. Scale bars: 500 µm upper, 100 µm lower. (**b**) Representative FACS analysis of calcein-AM-treated C2C12 myoblasts (upper) and cSCs 7 days after isolation. The lower, middle, and upper 10–15% of the population were sorted as Calcein^low^, Calcein^middle^, and Calcein^high^ cSCs, respectively. *n* > 3. (**c**) Expression of *Pax7*, *Myf5*, *MyoD*, *Myogenin*, and *Desmin* in Calcein^low^, Calcein^middle^, and Calcein^high^ cSCs. *n* = 6–8. (**d**) Representative bright-field images of Calcein^low^, Calcein^middle^, and Calcein^high^ cSCs 0 and 3 days after FACS sorting. *n* = 4. Scale bar: 200 µm. (**e**) Quantitative analysis of numbers of Calcein^low^, Calcein^middle^, and Calcein^high^ cSCs. *n* = 4. (**f**) Representative images of BrdU-positive Calcein^low^, Calcein^middle^, and Calcein^high^ cSCs. *n* = 5. Scale bar: 200 µm. (**g**) Quantitative analysis of BrdU-positive cells. *n* = 5. (**h**) Representative images of Dystrophin-positive myofibers in *mdx* mice transplanted with Calcein^low^, Calcein^middle^, and Calcein^high^ cSCs, respectively. Scale bar: 100 µm. (**i**) Quantitative analysis of numbers of Dystrophin-positive fibers in transplanted tibialis anterior/extensor digitorum longus muscles. *n* = 3. Error bars indicate SEM. *P < 0.05, **P < 0.01, ***P < 0.001 by ANOVA with Tukey–Kramer test.

In this study, we found a relationship between SC heterogeneity and intracellular esterase activity. Calcein-AM is a widely used cell-staining reagent that has a chemical structure of AM. We treated primary cultured SCs (cSCs) with calcein-AM 7 days after isolation by single fiber culture, when undifferentiated stem cells and differentiated myotubes existed simultaneously. We observed strong calcein fluorescence in differentiated myotubes (Fig. 1a, upper left). Interestingly, we also observed heterogeneous calcein fluorescence among undifferentiated round cells, suggesting a relationship between esterase activity and heterogeneity of cSCs (Fig. 1a, upper right and lower). We therefore separated subpopulation of cSCs based on calcein intensity by fluorescence-activated cell sorting (FACS). Dynamic range of calcein intensity in cSCs was wider than C2C12 mouse muscle cell line, indicating the heterogeneous esterase activity in cSCs (Fig. 1b upper). The lower, middle, and upper 10–15% of the population were defined as Calcein^low^, Calcein^middle^ and Calcein^high^ cSCs, respectively (Fig. 1b lower). The expression of *Pax7* was 3-fold lower in Calcein^high^ cSCs compared with the other two groups (Fig. 1c). Consistent with this, expression of *Myogenin* and *Desmin* were also higher in Calcein^high^ cSCs; notably, expression of *Myogenin* was 10-fold higher in Calcein^high^ cSCs compared with the other two groups, suggesting that differentiating or differentiated myocytes were enriched in Calcein^high^ cSCs. Calcein^high^ cSCs also showed 3-fold lower and 4-fold higher expression levels of *Myf5* and *MyoD* compared with Calcein^low^ cSCs, respectively. We assessed cell proliferation ability by analyzing time-dependent changes of the cell numbers (Fig. 1d,e). Proliferation of Calcein^high^ cSCs was lower than that of the other two groups. Calcein^low^ cSCs showed intermediate proliferation. Furthermore, the percentage of bromodeoxyuridine (BrdU)-positive cells was highest in Calcein^middle^ cSCs and lowest in Calcein^high^ cSCs (Fig. 1f,g). We transplanted these different subpopulations into *mdx* mice, a mouse model of Duchenne muscular dystrophy, and counted the numbers of Dystrophin-positive engrafted fibers. We observed the highest number of Dystrophin-positive fibers in Calcein^low^ cSCs-transplanted *mdx* mice (Fig. 1h,i). Overall, these results indicated that the esterase activity was increased with the differentiation of cSCs, and: 1) differentiating, non-proliferating cells were enriched in Calcein^high^ cSCs; 2) vigorously growing cells were enriched in Calcein^middle^ cSCs; and 3) relatively undifferentiated cSCs, which showed low proliferation and high transplantation efficiency, were enriched in Calcein^low^ cSCs.

Expression of *Myogenin* in Calcein^middle^ cSCs was 2 to 3 times higher than that in the Calcein^low^ cSCs, though no difference in expression of *Pax7* was detected between these subpopulations (Fig. 1c). Together with the differences in proliferation ability (Fig. 1d–g) and transplantation efficiency (Fig. 1h,i), these results implied that Calcein^low^ cSCs had the highest stemness. We analyzed the molecular signature of these cells by genome-wide gene expression analysis (Supplementary Table 1). Several genes related to muscle development and structural components (i.e., myofibril, muscle contraction) were enriched in Calcein^high^ cSCs compared with Calcein^low^ cSCs (Supplementary Table 2). Interestingly, genes related to muscle development and structural components were also enriched in Calcein^middle^ cSCs compared with Calcein^low^ cSCs, further supporting the idea that undifferentiated stem cells were enriched in the Calcein^low^ fraction.

**Figure 2:**
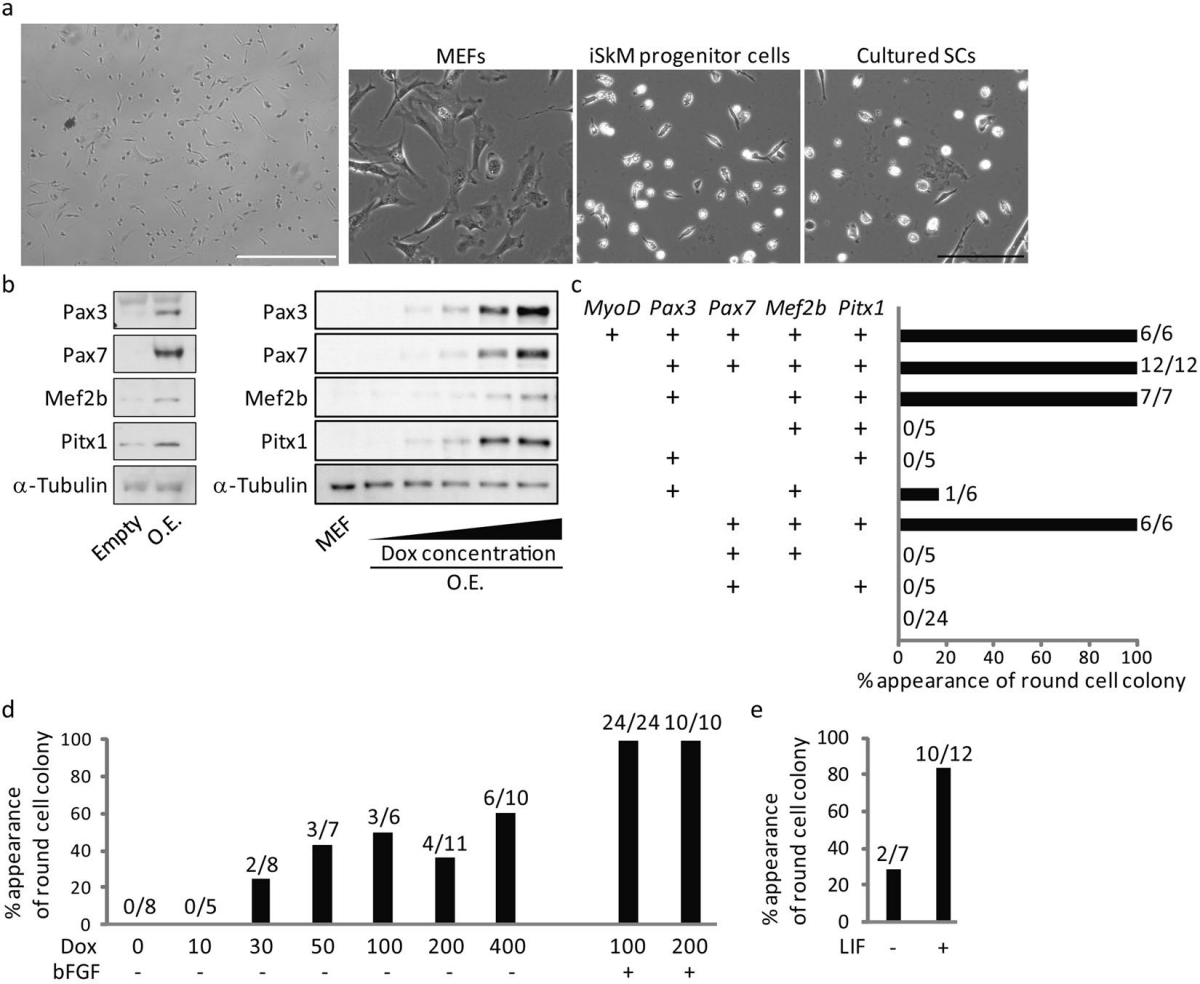
Generation of iSkM progenitor cells by exogenous expression of Calcein^low^ SCs-enriched TFs from MEFs. (**a**) Representative morphology of iSkM progenitor cells generated from MEFs. Low-power field of iSkM progenitor cells (left; scale bar: 500 µm) and high-power field of MEFs, iSkM progenitor cells, and cultured SCs (right; scale bar: 200 µm). *n* > 3. (**b**) Representative western blot showing overexpression of Pax3, Pax7, Mef2b, and Pitx1 by retrovirus (left) and dox-inducible lentivirus vectors, respectively (dox concentrations was 0, 1, 3, 10, 30 ng/ml). O.E., overexpression. *n* = 3. (**c**) Analysis of TFs essential for generating iSkM progenitor cells by retrovirus vector. (**d**) The incidence of iSkM progenitor cells by dox-inducible lentivirus vector. (**e**) Treatment with LIF enhanced the production efficiency of iSkM progenitor cells. Number of established lines/number of trials is indicated on the upper part of bar in (**c**,**d** and **e**).

Given these results, we hypothesized that TFs enriched in Calcein^low^ cSCs include those with the ability to maintain the undifferentiated state, which might have the potential to induce non-muscle cells, such as fibroblasts, into the myogenic lineage. We investigated TFs (*Meox1*, *Meox2*, *Mef2b*, *Twist1*, *Twist2*, *Pitx1*, and *Hoxc12*) with higher expression levels in Calcein^low^ cSCs compared with Calcein^middle^ cSCs (Supplementary Fig. 2), and exogenously expressed these TFs in mouse embryonic fibroblasts (MEFs), together with *Pax3*, *Pax7*, and *MyoD*, using retrovirus. We observed colonies of round cells with morphology similar to cSCs (Fig. 2a), and designated these as iSkM progenitor cells. We removed the overexpressed TFs one by one to identify which TFs were essential, and identified either *Pax3*, *Mef2b*, and *Pitx1*, or *Pax7*, *Mef2b*, and *Pitx1* as essential TFs for the induction of iSkM progenitor cells from MEFs (Fig. 2b,c), while *MyoD* was not required. We quantified the number and size of colonies. Both number and size of colonies was much higher in *Pax7*-expressing lines compared with that in *Pax3*-expressing lines (Supplementary Table 3). We also used doxycycline (dox)-inducible lentivirus in which expression of exogenes was upregulated by addition of dox with stable expression of orange fluorescent protein Kusabira-Orange^20,21^ (Fig. 2b). Dox-induced expression of these TFs resulted in formation of iSkM progenitor cells in a concentration-dependent manner (Fig. 2d). Furthermore, leukemia inhibitory factor (LIF) and basic fibroblast growth factor (bFGF), well-known growth factors that inhibit differentiation of SCs^22,23^, enhanced production efficiency of iSkM progenitor cells (Fig. 2d,e). We defined iSkM progenitor cells that were established from each dish as a cell line, rather than a clone, because each line was composed of a heterogeneous cell population, as described below.

**Figure 3:**
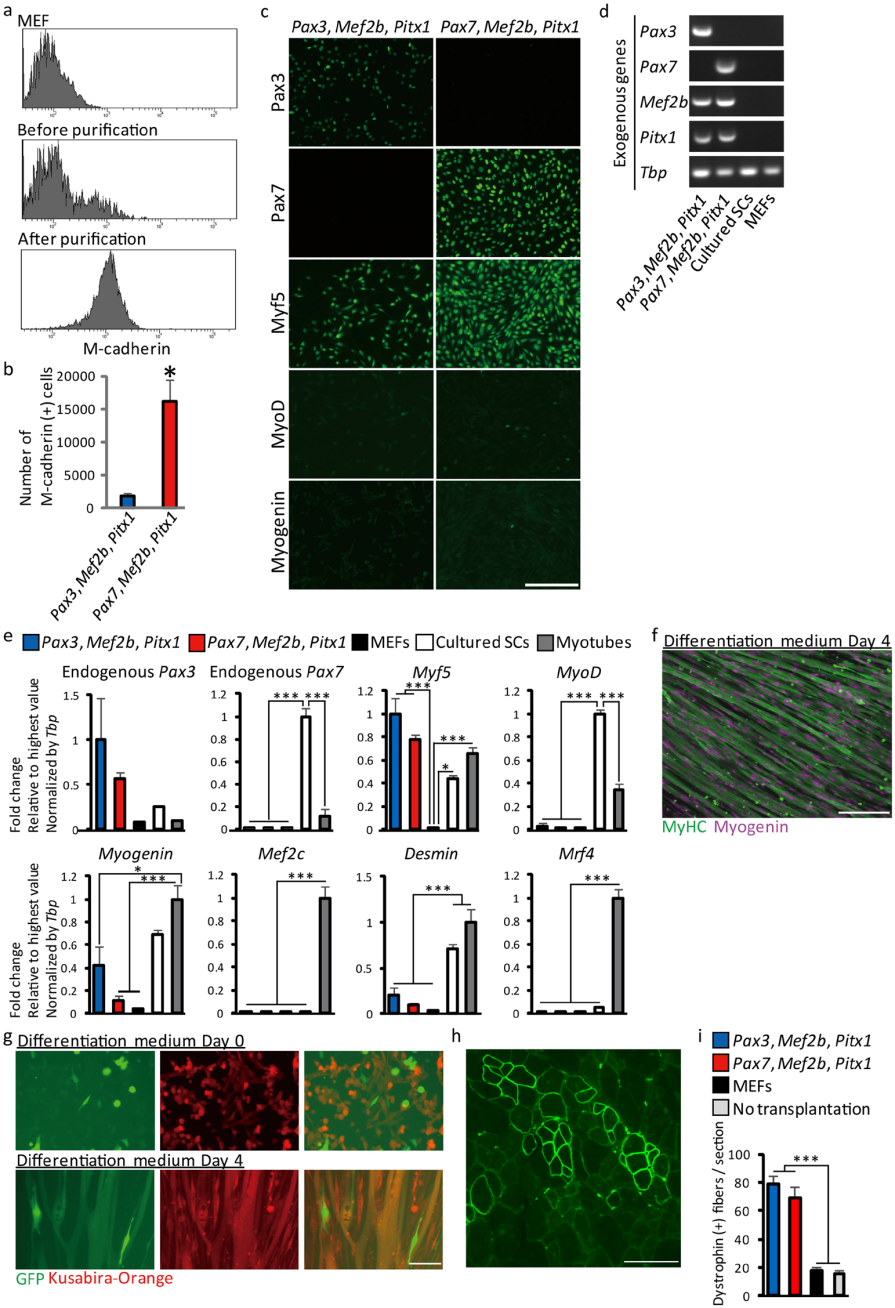
Myogenic property of iSkM progenitor cells generated from MEFs. (**a**) Representative expression of M-cadherin analyzed by FACS in MEFs (upper), mixture of MEFs and iSkM progenitor cells (middle), and purified iSkM progenitor cells (lower). *n* > 3 for each group. (**b**) Numbers of M-cadherin-positive cells generated by exogenous expression of *Pax3*, *Mef2b*, and *Pitx1* or *Pax7*, *Mef2b*, and *Pitx1*. *n* = 6. (**c**) Immunocytochemical analysis of Pax3, Pax7, Myf5, MyoD, and Myogenin in iSkM progenitor cells. Scale bar: 200 µm. *n* = 6. (**d**) Expression of exogenous genes in iSkM progenitor cells. *n* = 6. (**e**) Endogenous expression of several muscle marker genes in iSkM progenitor cells, MEFs, cultured SCs, and myotubes. *n* = 6. (**f**) Representative immunostaining of MyHC and myogenin in iSkM cells 4 days after induction of differentiation. *n* > 6. Scale bar: 200 µm. (**g**) Co-culture of GFP–cSCs (GFP: green) and iSkM progenitor cells (Kusabira-Orange: red) showing formation of GFP/Kusabira-Orange-double-positive myotubes. *n* > 6. Scale bar: 200 µm. (**h**) Representative images of Dystrophin-positive myofibers in iSkM progenitor cell from MEFs-transplanted *mdx* mice. Scale bar: 100 µm. (**i**) Quantitative analysis of numbers of Dystrophin-positive fibers in transplanted tibialis anterior/extensor digitorum longus muscles. *n* = 4–6. Error bars indicate SEM. ^*^P < 0.05, ^**^P < 0.01, ^***^P < 0.001 by Student’s *t*-test in (**b**), or by ANOVA with Tukey–Kramer test in (**e**,**i**).

To purify and characterize the iSkM progenitor cells derived from MEFs, we analyzed the several surface markers by FACS. iSkM progenitor cells were purified based on their expression of M-cadherin, a SC and myogenic cell marker^24^ (Fig. 3a), and were used in subsequent experiments. Expression of CD3e, CD11b, CD31, CD34, CD36, CD45, CD45R, CD68, CD93, CD135, Flk-1, PDGFRα, Ly-6G and TER-119 in iSkM progenitor cells was comparable to that in MEFs (Supplementary Fig. 3). The number of M-cadherin-positive cells was significantly higher in *Pax7*-expressing lines, though the incidence of colonies in *Pax3*-expressing lines was comparable to that in *Pax7*-expressing lines (Fig. 3b), suggesting the overlapping but distinct function between Pax3 and Pax7^25^. Myf5 was expressed in these cells, though MyoD and Myogenin were rarely detected (Fig. 3c). In addition to exogenous expression of *Pax3* or *Pax7*, *Mef2b*, and *Pitx1* (Fig. 3d), endogenous expression of *Myf5*, but not *MyoD*, was comparable to cSCs (Fig. 3e). Endogenous expression of *Pax7* was not observed. Endogenous expression of *Pax3* was slightly higher than cSCs, though its differences were not statistically significant. Together with the low expression level of *Pax3* in limb muscle^25^, these results suggested that iSkM progenitor cells maintained their myogenic properties by endogenous expression of *Myf5* and exogenous expression of *Pax3* or *Pax7*. Expression levels of the differentiation markers, *Myogenin*, *Mef2c*, *Mrf4*, and *Desmin* in iSkM progenitor cells were lower than those in differentiated myotubes (Fig. 3e). Bisulfite genomic sequencing demonstrated that the promoter regions of *Myf5* were demethylated overall in iSkM progenitor cells (Supplementary Fig. 4), while both demethylated and methylated sites were observed simultaneously in some cell lines, suggesting the existence of a heterogeneous population. We further evaluated the myogenic potential of iSkM progenitor cells by analyzing their ability to differentiate into myotubes *in vitro*. Multi-nucleated myosin heavy chain (MyHC)-positive myotubes were detected 4 days after induction with removal of dox (Fig. 3f). Co-culture of iSkM progenitor cells with cSCs isolated from GFP mice resulted in the formation of both Kusabira-Orange- and GFP-positive myotubes (Fig. 3g), suggesting that iSkM progenitor cells formed myotubes by fusing with endogenous muscle cells. Although brown adipocytes and skeletal muscle cells originate from the same *Myf5*-positive cells, iSkM progenitor cells did not differentiate into white or brown adipocytes^26^ (Supplementary Fig. 5). We transplanted iSkM progenitor cells derived from MEFs into *mdx* mice, and observed a significant increase in Dystrophin-positive myofibers (Fig. 3h,i).

**Figure 4:**
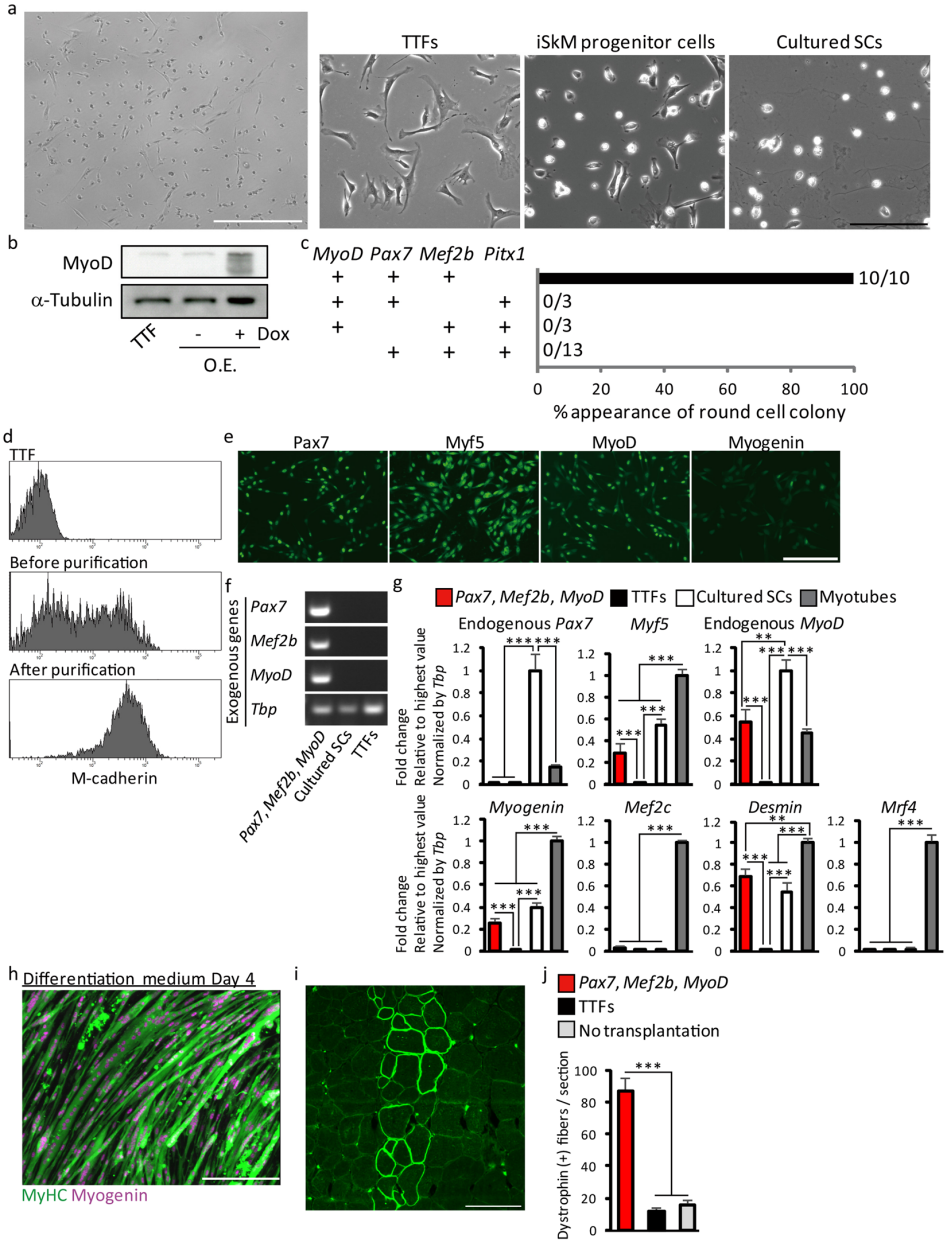
Induction of skeletal muscle progenitor cells from adult fibroblasts with *Pax7*, *Mef2b*, and *MyoD*. (**a**) Representative morphology of iSkM progenitor cells generated from TTFs. Low-power field of iSkM progenitor cells (left; scale bar: 500 µm) and high-power field of TTFs, iSkM progenitor cells, and cultured SCs (right; scale bar: 200 µm). *n* > 3. (**b**) Representative western blot for exogenous expression of MyoD by dox-inducible lentivirus vector (dox concentration was 0 or 100 ng/ml). O.E., overexpression. *n* = 3. (**c**) Analysis of TFs essential for generation of iSkM progenitor cells from TTFs. (**d**) Representative expression of M-cadherin analyzed by FACS in TTFs (upper), mixture of TTFs and iSkM progenitor cells (middle), and purified iSkM progenitor cells (lower). *n* > 4 for each group. (**e**) Immunocytochemical analysis of Pax3, Pax7, Myf5, MyoD, and Myogenin in iSkM progenitor cells. Scale bar: 200 µm. *n* > 4. (**f**) Expression of exogenous genes in iSkM progenitor cells. *n* = 6. (**g**) Endogenous expression of several marker genes in iSkM progenitor cells, TTFs, cultured SCs, and myotubes. *n* = 6–8. (**h**) Representative immunostaining of MyHC and Myogenin in iSkM cells 4 days after induction of differentiation. *n* > 6. Scale bar: 200 µm. (**i**) Representative images of Dystrophin-positive myofibers in iSkM progenitor cell-transplanted *mdx* mice. Scale bar: 100 µm. (**j**) Quantitative analysis of numbers of Dystrophin-positive fibers in transplanted tibialis anterior/extensor digitorum longus muscles. *n* = 4–9. Error bars indicate SEM. ^**^P < 0.01, ^***^P < 0.001 by ANOVA with Tukey–Kramer test in (**g**,**j**).

Round iSkM progenitor cells were induced from adult tail-tip fibroblasts (TTFs) by exogenous expression of *Pax7*, *Mef2b*, and *Pitx1*. However, we failed to expand these cells, suggesting that exogenous expression of these TFs was not sufficient to induce stable iSkM progenitor cells from adult fibroblasts (data not shown). Similar difficulties in inducing cardiomyocytes from TTFs, which were more resistant to reprogramming, have also been reported^27^. Therefore, we expressed these factors exogenously with *MyoD*, the first identified reprogramming factor to induce myocytes from fibroblasts^4^, which was not required for induction of iSkM progenitor cells from MEFs. iSkM progenitor cells were induced from TTFs by exogenous expression of *Pax7*, *Mef2b*, *Pitx1*, and *MyoD* (Fig. 4a). *Pitx1* was not required in the presence of *MyoD*, suggesting that *Pitx1* and *MyoD* have overlapping functions (Fig. 4b,c). iSkM progenitor cells derived from TTFs were positive for M-cadherin (Fig. 4d), and immunocytochemistry revealed that these cells expressed Pax7, Myf5, and MyoD, but not Myogenin (Fig. 4e). Gene expression analysis showed the endogenous expression of *Myf5* and *MyoD* in iSkM progenitor cells derived from TTFs (Fig. 4f,g). These cells formed myotubes *in vitro* (Fig. 4h). Finally, we transplanted iSkM progenitor cells derived from TTFs into *mdx* mice, and observed a significant increase in Dystrophin-positive myofibers (Fig. 4i,j). Overall, we achieved direct reprogramming of fibroblasts into skeletal muscle progenitor cells by defined factors enriched in an undifferentiated subpopulation of cSCs (Supplementary Fig. 6).

## Discussion

Loss of the undifferentiated state and subsequent differentiation are associated with intracellular metabolic systems such as anaerobic glycolysis and oxidative phosphorylation in SCs and hematopoietic stem cells^28^. In this study, we identified esterase activity as a novel hierarchical marker of cSCs. Further studies are needed to determine the molecular mechanisms linking esterase activity or its metabolites to heterogeneity of cSCs. The relationship between esterase activity and stemness in more superior stem cells, such as quiescent SCs and other tissue stem cells, is also worthy of consideration.

We identified the combination of *Pax3*, *Mef2b*, and *Pitx1* or *Pax7*, *Mef2b*, and *Pitx1* in embryonic fibroblasts, and *Pax7*, *Mef2b* and *MyoD* in adult fibroblasts as essential TFs for inducing skeletal muscle progenitor cells. The spatiotemporal pattern of *Mef2b* expression in developing skeletal muscle lineages differs from that of other *Mef2* genes that are required for adult muscle regeneration^29,30^. In accordance with these previous studies, we found that only *Mef2b* was upregulated in Calcein^low^ cSCs, while others were upregulated in Calcein^high^ cSCs (Supplementary Table 1), suggesting the unique character of *Mef2b*. In addition to *Mef2b*, the other Calcein^low^ cSCs-enriched TFs included noteworthy genes from the past findings. *Pitx1* was required for hindlimb patterning and development by upregulating *Tbx4*^31,32^. *Meox1* and *Meox2* were expressed in developing limb buds, and their disruption resulted in impaired expression of *Pax3*, *Pax7*, and *Myf5* in mouse embryos^33,34^. Twist, in co-operation with Notch, negatively regulated muscle differentiation in *Drosophila*^35,36^. Analysis of Calcein^low^ cSCs-enriched genes other than TFs may help to elucidate the underlying system for the maintenance of heterogeneous populations and their stemness in SCs.

Distinct from the exogenous expression of *MyoD* alone^4^, the induction of iSkM cells included a progenitor-cell state, allowing these cells to be purified and their proliferation maintained. Importantly, iSkM progenitor cells did not show high endogenous expression of *Pax3* or *Pax7* (Figs 3e and 4g), suggesting that iSkM progenitor cells were not “induced muscle stem cells”. Induction of these muscle stem cells might require upstream TFs responsible for inducing the endogenous expression of *Pax3* or *Pax7*. Because overexpression of *MyoD* exerts anti-proliferation effects, requirement of *MyoD* to generate proliferating muscle progenitor cells from TTFs seems to be controversial. As proliferating cSCs express *MyoD*, some TFs, such as Pax7, might inhibit the anti-proliferating effects of *MyoD*. Overall, we investigated the molecular background of cSC subpopulations and identified TFs essential for inducing skeletal muscle progenitor cells from fibroblasts. These results may contribute to the development of new therapeutics for muscle degenerative diseases, such as Duchenne muscular dystrophy.

## Materials and Methods

### Animals

Twelve- to sixteen-week-old male C57BL/6 mice were purchased from Nihon CREA. C57BL/6-GFP transgenic mice were kindly provided by Dr. Masaru Okabe (Osaka University, Osaka, Japan). C57BL/6-mdx mice were a kind gift from Dr. Toshikuni Sasaoka (National Institute for Basic Biology, Aichi, Japan). All mice were housed at the institutional animal facility. All of the animal procedures were approved by the Experimental Animal Care and Use Committee at the National Center of Neurology and Psychiatry (NCNP, Tokyo, Japan). All of the experimental methods were performed in accordance with approved guidelines.

### Single muscle fiber preparation

For preparation of primary SCs, extensor digitorum longus (EDL) muscles were isolated from 12–16-week-old male C57BL/6 and C57BL/6-GFP mice and dissociated with type 1 collagenase (Worthington), as described previously^18^. Briefly, isolated EDL muscles were incubated in 0.2% type 1 collagenase/DMEM for 80–90 min. Dissociated muscles were unraveled by gentle pipetting. Single muscle fibers were isolated and plated on Matrigel (Matrigel™-Growth Factor Reduced, BD Biosciences)-coated dishes.

### Cell culture

Isolated muscle fibers with primary SCs were cultured in DMEM (high glucose, sodium pyruvate, and GlutaMAX supplement; Thermo Fisher Scientific) supplemented with 20% fetal bovine serum (FBS), 1% chick embryo extract (CEE) (US Biological), and 1% penicillin-streptomycin (PS) (Thermo Fisher Scientific) at 37 °C with 5% CO2. Established iSkM progenitor cells were cultured in DMEM (high glucose, sodium pyruvate, and GlutaMAX supplement) supplemented with 10% FBS, 1% CEE, 5 ng/ml basic fibroblast growth factor (Cell Signaling), 5 ng/ml LIF (PROSPEC), 100 ng/ml dox (LCK Laboratories), and 1% PS. All plastic dishes used for cSCs and iSkM progenitor cells were coated with Matrigel. C2C12, 3T3-L1, MEFs, TTFs, Plat-E, and HEK293T cells were cultured in DMEM (high glucose; Wako) supplemented with 10% FBS and 1% PS at 37 °C with 5% CO2. The medium was changed every 2 days.

### Acetoxymethyl ester chemicals treatment

Primary cSCs 4 or 7 days after isolation were treated with 50 nM calcein-AM (Dojindo) in DMEM (high glucose, sodium pyruvate, and GlutaMAX supplement) supplemented with 20% FBS, 1% CEE, and 1% PS at 37 °C with 5% CO2 for 30 min. C2C12 myoblasts were treated with 50 nM calcein-AM in DMEM (high glucose) supplemented with 10% FBS and 1% PS at 37 °C with 5% CO2 for 30 min. C2C12 myotubes 4 days after induction of differentiation were treated with 1 μM BCECF-AM (DOJINDO), 1 μM CFSE (DOJINDO), 1 μM FDA (DOJINDO) or 1 μM calcein-AM in DMEM (high glucose) supplemented with 2% horse serum and 1% PS at 37 °C with 5% CO2 for 30 minutes.

### FACS analysis

Calcein-AM-treated cSCs 4 or 7 days after isolation were dissociated by 0.05% trypsin/EDTA (Thermo Fisher Scientific) at 37 °C with 5% CO2 for 5 min. Cell sorting was performed on a FACS ARIA (BD Biosciences). The lower, middle, and upper 10–15% populations of calcein-AM-treated cSCs were defined as Calcein^low^, Calcein^middle^, and Calcein^high^ cSCs, respectively, based on the fluorescence intensity of calcein. For surface-marker analysis and purification by M-cadherin antibody, cells were dissociated in Cell Dissociation Buffer (Thermo Fisher Scientific) at 37 °C with 5% CO2 for 30 min, followed by vigorous pipetting. The antibodies used for FACS analysis were: fluorescein isothiocyanate (FITC)-anti-CD31 (1:200, Clone: 390; BD Biosciences), FITC-anti-CD34 (1:200, Clone: RAM34; BD Biosciences), FITC-anti-CD45 (1:200, Clone: 30-F11; BD Biosciences), Alexa488-anti-CD36 (1:200, Clone: HM36; BioLegend), FITC-anti-CD93 (1:200, Clone: AA4.1; BD Biosciences), FITC-anti-CD68 (1:200, Clone: FA-11; BD Biosciences), APC-anti-CD140 (1:200, Clone: APA5; BD Biosciences), biotin-anti-Ly-6G/Ly-6C (1:200, Clone: RB6-8C5; BioLegend), biotin-anti-CD3e (1:200, Clone: 145-2C11; BioLegend), biotin-anti-CD11b (1:200, Clone: M1/70; BioLegend), biotin-anti-CD45R/B220 (1:200, Clone: RA3-6B2; BioLegend), biotin-anti-TER-119/erythroid cells (1:200, Clone: TER-119; BioLegend), APC-anti-VCAM1 (1:200, Clone: 429 (MVCAM.A); BioLegend), APC-anti-FLK-1 (1:200, Clone: Avas 12alpha1; BD Biosciences), APC-anti-CD135 (1:200, Clone: A2F10; BioLegend), anti-M-cadherin (1:400, Clone: 5/M-Cadherin; BD Biosciences), and APC-anti-mouse IgG (1:200, Clone: Poly4053; BioLegend).

### Proliferation assay

After FACS sorting, 1.0 × 10^4^ Calcein^low^, Calcein^middle^, and Calcein^high^ cSCs, respectively, 4 days after isolation were seeded onto 24-well plates and the numbers of cells per field were counted every day. For BrdU assay, cSCs 4 days after isolation were treated with 10 μM BrdU (Sigma Aldrich) in DMEM (high glucose, sodium pyruvate, and GlutaMAX supplement) supplemented with 20% FBS, 1% CEE, and 1% PS at 37 °C with 5% CO2 for 2 h. The cells were then treated with 50 nM calcein-AM followed by FACS sorting. Sorted Calcein^low^, Calcein^middle^, and Calcein^high^ cSCs were fixed with 4% paraformaldehyde/phosphate-buffered saline (PBS). After washing, fixed cells were treated with 0.1% Triton X-100/PBS for 10 min for permeabilization, followed by 2 N HCl for 30 min at room temperature, blocking with 5% goat serum (Cedarlane) in 2% bovine serum albumin (BSA)/PBS for 15 min, and incubation with anti-BrdU (1:400, clone: BU1/75 (ICR1); AbD Serotec) in 2% BSA/PBS at 4 °C overnight. After washing, the cells were incubated with Alexa Fluor 488-labelled secondary antibody (1:1000; Thermo Fisher Scientific) in 1% BSA/PBS. After several washings, nuclei were stained with DAPI (Dojindo). Immunofluorescent-staining images were evaluated by fluorescence microscopy (Olympus IX71), and the percentage of BrdU-positive cells was counted.

### RNA isolation and reverse transcription–polymerase chain reaction analysis

Total RNA was isolated using TRIzol (Invitrogen). Single-strand cDNA was synthesized using a QuantiTect Reverse Transcription Kit (Qiagen) and conventional polymerase chain reaction (PCR) was performed using ExTaq (Takara). For quantitative reverse transcription (qRT)-PCR, the expression level of each gene was evaluated using SYBR Premix Ex Taq II (Takara) on a MyiQ single-color system (Bio-Rad). The primer sequences for RT-PCR were as follows: endogenous Pax7 forward: 5′-gcccacagaacctgtcactc-3′, reverse: 5′-ggacttgaaagcttggtgctct-3′, MyoD forward: 5′-tgagccttgcacacctaagcc-3′, reverse: 5′-ctccgcaagctgtggggaaa-3′, Myf5 forward: 5′-tctctcccgatgatcactcct-3′, reverse: 5′-tctgcccagcttgtctttcc-3′, Myogenin forward: 5′-tgtgcacatctgttctagtctc-3′, reverse: 5′-gctttggaaccggatagctc-3′, Mef2c forward: 5′-cggtgtcgtcagttgtatgg-3′, reverse: 5′-tgcagtagatatgcggcttg-3′, Mrf4 forward: 5′-aagtgtttcggatcattccag-3′, reverse: 5′-aaatactgtccacgatggaag-3′, Desmin forward: 5′-tggaataccgacaccagatcca-3′, reverse: 5′-ctgcctcatcagggagtcgtt-3′, Mef2b forward: 5′-ctccacagagttcccgaagt-3′, reverse: 5′-tccttgggctttattgatgcag-3′, Pitx1 forward: 5′-tacgaggacgtgtacgct-3′, reverse: 5′-tgaagaaggtaaagctcttggt-3′, Twist1 forward: 5′-gcggccaggtacatcgactt-3′, reverse: 5′-acatagctgcagcttgccatct-3′, Twist2 forward: 5′-aagatccagacgctcaag-3′, reverse: 5′-ctggtcatcttattgtccatc-3′, Meox1 forward: 5′-cataccccgacttctctgcttc-3′, reverse: 5′-gctgctcgttgaagattcgct-3′, Meox2 forward: 5′-cagacaggggactcactagca-3′, reverse: 5′-tcctgagaatggagctggtctt-3′, Hoxc12 forward: 5′-agccgtattcgaagttgcag-3′, reverse: 5′-ttcaagcggtccgagagt-3′, exogenous universal: 5′-cahhctttaaaggaacca-3′, exogenous Pax3: 5′-tgtcacctgcttgggttt-3′, exogenous Pax7: 5′-ccatggtgggccatttccact-3′, exogenous Mef2b: 5′-ctcttcagtgtctgaagg-3′, exogenous Pitx1: 5′-tggcttgtgaagtgagtg-3′, and TATA-binding protein (Tbp) forward: 5′-cagcctcagtacagcaatcaac-3′, reverse: 5′-taggggtcataggagtcattgg-3′. For RT–qPCR, the expression level of each gene was normalized to that of Tbp.

### Microarray analysis

Total RNA was isolated from 1.0 × 10^5^ Calcein^low^, Calcein^middle^, and Calcein^high^ cSCs 7 days after isolation, using TRIzol. RNA samples were analyzed using the Agilent

SurePrint G3 Mouse GE 8 × 60 K microarray platform, by Medical & Biological Laboratories. Obtained data were analyzed by GeneSpring GX. All expression data were applied to 75^th^ percentile normalization. Genes defined as “not detected” in all three groups were removed from the analysis. Genes with expression levels >2-fold higher in Calcein^high^ compared with Calcein^low^ cSCs (total 2,653 genes), and >2-fold higher in Calcein^middle^ compared with Calcein^low^ cSCs (total 812 genes) were used for gene ontology analysis. More than 70 significant gene ontologies were identified, of which the top 14 ontologies are shown in Supplementary Table 2.

### Immunocytochemistry

The cells were fixed with 4% paraformaldehyde/PBS for 10 min at room temperature. After washing, fixed cells were treated with 0.1% Triton X-100/PBS for 10 min for permeabilization, blocked for 15 min with 6% skim milk (Megmilk Snow Brand)/PBS for anti-Myf5 antibody, or 5% goat serum in 2% BSA/PBS for the other antibodies. The cells were then incubated with anti-Pax3 (1:80; Developmental Studies Hybridoma Bank), anti-Pax7 (1:80, sc-81648; Santa Cruz), anti-MyoD (1:400, sc-32758; Santa Cruz), anti-myogenin (1:1000, sc-12732; Santa Cruz), or anti-MyHC antibody (1:500, MAB4470, Clone: MF20; R&D Systems) in 2% BSA/PBS, or anti-Myf5 antibody (1:200, sc-302; Santa Cruz) in 6% skim milk/PBS at 4 °C overnight, followed by incubation with Alexa Fluor 488- or Alexa Fluor 647-labeled secondary antibodies (1:1000; Thermo Fisher Scientific) in 2% BSA/PBS. After several washings, nuclei were stained with DAPI (Dojindo). Immunofluorescent staining images were evaluated by fluorescence microscopy (Olympus).

### Immunohistochemistry

Transplanted tibialis anterior (TA) and EDL muscles were cut by cryostat into 8-μm cross sections and fixed with cooled acetone for 10 min. After air-drying, the sections were blocked with M.O.M. Mouse Ig Blocking reagent (Vector M.O.M. Immunodetection Kit; Vector) for 1 h, followed by 5% goat serum in 1% BSA/PBS for 15 min, and then incubated with anti-Dys2 antibody (1:100, Clone: Dy8/6C5; Leica) in M.O.M. diluent at 4 °C overnight. The sections were then incubated with Alexa Fluor 488-labeled secondary antibodies (1:1000; Thermo Fisher Scientific) in M.O.M. diluent. After several washings, nuclei were stained with DAPI (Vector). Immunofluorescent staining images were evaluated by fluorescence microscopy (BZ-9000; Keyence), and dystrophin-positive fibers were counted.

### Plasmid construction

The coding regions of target genes were amplified from the cDNAs of primary cSCs by RT-PCR 7 days after isolation to generate pMXs-puro-Pax3, pMXs-puro-Pax7, pMXs-IG-MyoD, pMXs-IG-Mef2b, pMXs-IG-Pitx1, pMXs-IG-Twist1, pMXs-IG-Twist2, pMXs-IG-Hoxc12, pMXs-IG-Meox1 and pMXs-IG-Meox2. CSIV-TRE-Pax3-CMV-KT, CSIV-TRE-Pax7-CMV-KT, CSIV-TRE-Mef2b-CMV-KT, CSIV-TRE-Pitx1-CMV-KT, and CSIV-TRE-MyoD-CMV-KT were generated by cloning the coding regions of target genes into pENTR1A vector. These plasmids were recombined with the CSIV-TRE-RfA-CMV-KT vector by LR reaction (Thermo Fisher Scientific).

### Preparation of retrovirus and lentivirus

Retrovirus and lentivirus were prepared as described previously, with minor modifications^37^. Briefly, Plat-E packaging cells were used for retrovirus preparation. pMXs-based retrovirus vectors were transfected into 20–30% confluent Plat-E cells using Fugene 6 (Roche) according to the manufacturer’s recommendations, with minor modifications. Nine micrograms of plasmid vector were diluted in 300 μl of opti-MEM (Thermo Fisher Scientific), and 27 μl of Fugene 6 was then added to the mixture. After 15 min incubation at room temperature, the mixture was added to Plat-E cells in DMEM at 37 °C with 5% CO2 overnight. After changing the medium to DMEM supplemented with 10% FBS and 1% PS, the supernatants were collected for 4 days, and filtered with a Steriflip-HV, 0.45 μM filter (Millipore) to remove debris. The filtered supernatants were concentrated by polyethylene glycol precipitation. HEK293T cells were used for lentivirus preparation, similar to the procedure for retrovirus preparation, with minor modifications. Briefly, 4.2 μg CSIV-TRE-target gene-CMV-KT, 2.4 μg pCMV-VSV-G-RSV-Rev, and 2.4 μg pCAG-HIVgp were diluted in 300 μl opti-MEM, followed by the addition of 27 μl of Fugene 6. After 15 min incubation at room temperature, the mixture was added to 50–70% confluent HEK293T cells in DMEM at 37 °C with 5% CO_2_ overnight. After changing the medium to DMEM supplemented with 10% FBS, 10 μM forskolin (Wako), and 1% PS, the supernatants were collected for 4 days, filtered through a Steriflip-HV, 0.45 μM filter, and concentrated by polyethylene glycol precipitation.

### Preparation of MEFs

Primary MEFs was isolated from 13.5-day-pregnant C57BL/6 mice, as described previously^37^. Briefly, the head, limbs, tail, and viscera were removed from E13.5 embryos, and the remaining bodies were washed in PBS, minced, and incubated in 0.25% Trypsin/EDTA (Gibco) at 37 °C for 15–20 min. After trypsinization, an equal amount of DMEM containing 10% FBS and 1% PS was added, followed by gentle pipetting. The tissue/medium mixture was filtered through a 100 μm nylon Cell Strainer (BD Falcon). Filtered cells were collected by centrifugation and resuspended in fresh DMEM supplemented with 10% FBS and 1% PS, and seeded in plastic dishes. Attached MEFs were used.

### Preparation of TTFs

Primary TTFs were isolated from 12–16-week-old male C57BL/6 mice. Tail tips were washed in PBS, minced, and incubated in DMEM containing 1000 U/ml dispase I (Wako) and 2 mg/ml collagenase type 2 (Worthington) at 37 °C for 30 min. After digestion, an equal amount of DMEM containing 10% FBS and 1% PS was added, followed by gentle pipetting. The tissue/medium mixture was filtered through a 100 μm nylon Cell Strainer. The filtered cells were collected by centrifugation and resuspended in fresh DMEM supplemented with 10% FBS and 1% PS, and seeded in plastic dishes. Attached TTFs were used 4-5 days after incubation.

### Generation of iSkM progenitor cells

Prepared MEFs and TTFs were seeded at 2.0 × 10^5^ cells/6-well plate and 1.0 × 10^5^ cells/6-well plate, respectively. For MEFs, 24 h later, retrovirus-containing DMEM supplemented with 10% FBS, 8 μg/ml polybrene (Sigma Aldrich), and 1% PS was added and cultured overnight, or lentivirus-containing DMEM was added and cultured for 48 h. For TTFs, virus infection was started 10–12 h after seeding. After infection, the cells were passaged in 10-cm dishes and cultured for several days. GFP-positive or Kusabira-Orange-positive cells were purified by FACS, and approximately 2.5 × 10^5^ cells were then transferred to 10 cm dishes, and the formation of iSkM progenitor cells was observed within 2 weeks from the day of infection. The independent infection and sorting were performed to establish several lines.

### Western blotting

Western blotting was performed as described previously, with minor modifications^18^. Briefly, total protein was extracted in sample buffer containing 0.1% Triton X-100, 50 mM HEPES (pH 7.4), 4 mM EGTA, 10 mM EDTA, 15 mM Na4P2O7, 100 mM glycerophosphate, 25 mM NaF, 5 mM Na2VO4, and a complete protease inhibitor cocktail (Roche). The protein concentration was determined using Coomassie Brilliant Blue G-250 (Bio-Rad). Immediately prior to sodium dodecyl sulfate polyacrylamide gel electrophoresis (SDS-PAGE), the extracted protein solution was mixed with an equal volume of sample-loading buffer containing 30% glycerol, 5% 2-mercaptoethanol, 2.3% SDS, 62.5 mM Tris-HCl (pH 6.8), and 0.05% bromophenol blue, and the mixture was heated at 60 °C for 10 min. Eight micrograms of proteins were separated by SDS-PAGE and electrically transferred to a polyvinylidene difluoride membrane (Millipore). The blots were blocked by 6% skim milk/TBS for >1 h, followed by incubation with anti-Pax3 (1:100), anti-Pax7 (1:500), anti-Mef2b (1:50, sc-98595; Santa Cruz), anti-Pitx1 (1:500, sc-18922; Santa Cruz), anti-MyoD (1:100, sc-760; Santa Cruz), or anti-α-tubulin (1:4000, sc-12462; Santa Cruz) in 6% skim milk/TBS at 4 °C overnight. The blots were then incubated with horseradish peroxidase-conjugated secondary antibodies (1:2000) (anti-rabbit IgG, NA9340V; GE Healthcare, anti-goat IgG, 611620; Invitrogen, anti-mouse IgG, 115-036-062; Jackson Immuno Research). Signals were detected using an ECL™ Western Blotting Detection system (GE Healthcare).

### White and brown adipocyte differentiation assay

White and brown adipocyte differentiation was induced as described previously, with minor modifications^26^. For white adipocyte differentiation, cells were treated in DMEM containing 10% FBS, 10 μg/ml insulin (Wako), 2.5 μM dexamethasone (Sigma Aldrich), and 500 μM 3-isobutyl-1-methylxanthine (Sigma Aldrich). After 3 days, the medium was changed to DMEM containing 10% FBS and 10 μg/ml insulin for a further 5 days. For brown adipocyte differentiation, cells were treated in DMEM containing 10% FBS, 5 μg/ml insulin, 1 μM dexamethasone, 500 μM 3-isobutyl-1-methylxanthine, 125 nM indomethacin (Wako), 1 nM triiodothyronine (Merck), and 1 μM rosiglitazone (Wako). After 3 days, the medium was changed to DMEM containing 10% FBS, 5 μg/ml insulin, 1 nM triiodothyronine, and 1 μM rosiglitazone for a further 5 days. The medium was changed every day. After fixation by 4% paraformaldehyde/PBS, the cells were stained with Oil Red O and images were evaluated by microscopy (BZ-9000; Keyence).

### Bisulfite sequencing assay

Genomic DNA was purified using a NucleoSpin Tissue kit (Macherey-Nagel). Bisulfite-mediated conversion of cytosine to uracil was performed using a MethylEasy Xceed Rapid DNA Bisulphite Modification Kit (Human Genetic Signatures). The sequences from −927 to −529, −373 to +75, and +24 to +426 bp from Myf5 transcription start site were amplified by Takara EpiTaq HS (Takara), and DNA fragments were cloned into a pMD20-T vector. The primer sequences for bisulfite sequencing were as follows: Myf5 bisulfite 1 forward: 5′-gttggtagttgggatttatttgtaa-3′, reverse: 5′-caataactaaacttcctttctaaaatctaa-3′, Myf5 bisulfite 2 forward: 5′-aaagtttatgaaaatgagaagtaagtat-3′, reverse: 5′-aaacaaatacctattaaccaaaatc-3′, and Myf5 bisulfite 3 forward: 5′-aaagagttttaattttagttattgat-3′, reverse: 5′-aaactttacaaacccacataaaaca-3′. The primers were designed using MethPrimer^38^. Obtained sequences were analyzed using a quantification tool for methylation analysis (QUMA)^39^.

### Co-culture of GFP-cSCs and iSkM progenitor cells

GFP-cSCs and Kusabira-Orange-positive iSkM progenitor cells were co-cultured in 48-well plates. After replacement with the differentiation medium (DMEM high glucose, sodium pyruvate, and GlutaMAX supplement supplemented with 5% horse serum), fluorescent images were observed by fluorescence microscopy (Olympus). The medium was changed every day.

### Transplantation assay

Transplantation of cSCs and iSkM progenitor cells was performed as described previously, with minor modifications^40^. Briefly, 100 μl of 10 μM cardiotoxin (Sigma-Aldrich) was injected into the TA and EDL muscles of mdx mice 24 h before transplantation, to induce muscle regeneration. For cSC transplantation, 30 μl of cell suspension containing 3.0 × 10^4^ Calcein^low^, Calcein^middle^, or Calcein^high^ cSCs 7 days after isolation from C57BL/6 mice immediately after FACS sorting were injected directly into the TA/EDL muscles. For iSkM progenitor-cell transplantation, 30 μl of cell suspension containing 1.0 × 10^5^ cells were injected into the TA and EDL muscles. Each line was separately transplanted. The muscles were dissected 2 weeks later and frozen in isopentane cooled with liquid nitrogen.

### Statistical analysis

All values are expressed as mean ± standard error of the mean (SEM). The significance of differences was assessed by Student’s t-test or one-way analysis of variance (ANOVA), with differences among groups assessed by Tukey-Kramer post-hoc analysis. Probabilities less than 5% (^*^P < 0.05), 1% (^**^P < 0.01) or 0.1% (^***^P < 0.001), respectively, were considered to be statistically significant.

## Electronic supplementary material

Supplementary Table 1

Supplementary Figure
