# Author Correction: Direct reprogramming of fibroblasts into skeletal muscle progenitor cells by transcription factors enriched in undifferentiated subpopulation of satellite cells

**DOI:** 10.1038/s41598-018-24788-z

**Published:** 2018-04-25

**Authors:** Naoki Ito, Isao Kii, Noriaki Shimizu, Hirotoshi Tanaka, Shin’ichi Takeda

**Affiliations:** 1 Department of Molecular Therapy, National Institute of Neuroscience, National Center of Neurology and Psychiatry, Tokyo, 187-8502 Japan; 2 Department of Rheumatology and Allergy, IMSUT Hospital, The Institute of Medical Science, The University of Tokyo, Tokyo, 108-8639 Japan; 3 Pathophysiological and Health Science Team, Imaging Application Group, Division of Bio-Function Dynamics Imaging, Riken Center for Life Science Technologies, Hyogo, 650-0047 Japan; 4 Division of Rheumatology, Center for Antibody and Vaccine Therapy, IMSUT Hospital, The Institute of Medical Science, The University of Tokyo, Tokyo, 108-8639 Japan

Correction to: *Scientific Reports* 10.1038/s41598-017-08232-2, published online 14 August 2017

A correction to this article has been published and is linked from the HTML version of this paper. The error has been fixed in the paper.

