# Publisher Correction: Direct reprogramming of fibroblasts into skeletal muscle progenitor cells by transcription factors enriched in undifferentiated subpopulation of satellite cells

**DOI:** 10.1038/s41598-018-26910-7

**Published:** 2018-06-07

**Authors:** Naoki Ito, Isao Kii, Noriaki Shimizu, Hirotoshi Tanaka, Shin’ichi Takeda

**Affiliations:** 1 Department of Molecular Therapy, National Institute of Neuroscience, National Center of Neurology and Psychiatry, Tokyo, 187-8502 Japan; 2 Department of Rheumatology and Allergy, IMSUT Hospital, The Institute of Medical Science, The University of Tokyo, Tokyo, 108-8639 Japan; 3 Pathophysiological and Health Science Team, Imaging Application Group, Division of Bio-Function Dynamics Imaging, Riken Center for Life Science Technologies, Hyogo, 650-0047 Japan; 4 Division of Rheumatology, Center for Antibody and Vaccine Therapy, IMSUT Hospital, The Institute of Medical Science, The University of Tokyo, Tokyo, 108-8639 Japan

Correction to: *Scientific Reports* 10.1038/s41598-017-08232-2, published online 14 August 2017

The Author Correction published on 25^th^ April 2018 contains errors.

“A correction to this article has been published and is linked from the HTML version of this paper. The error has been fixed in the paper.”

should read:

“The original version of this Article contained a typographical error in the spelling of the author Shin’ichi Takeda, which was incorrectly given as Takeda Shin’ichi. This has now been corrected in the PDF and HTML versions of the Article.”

