# Supplementary material for: Direct reprogramming of fibroblasts into skeletal muscle progenitor cells by transcription factors enriched in undifferentiated subpopulation of satellite cells
Source: Sci Rep. 2017 Aug 14;7:8097. doi: 10.1038/s41598-017-08232-2 (PMC5556026; doi:10.1038/s41598-017-08232-2)
Supplement: Supplementary file 2 — Supplementary Figure [file 41598_2017_8232_MOESM2_ESM.pdf]

Direct reprogramming of fibroblasts into skeletal muscle progenitor cells by transcription factors enriched in undifferentiated subpopulation of satellite cells

Naoki Ito, Isao Kii, Noriaki Shimizu, Hirotoshi Tanaka and Shin'ichi Takeda

Supplementary Information

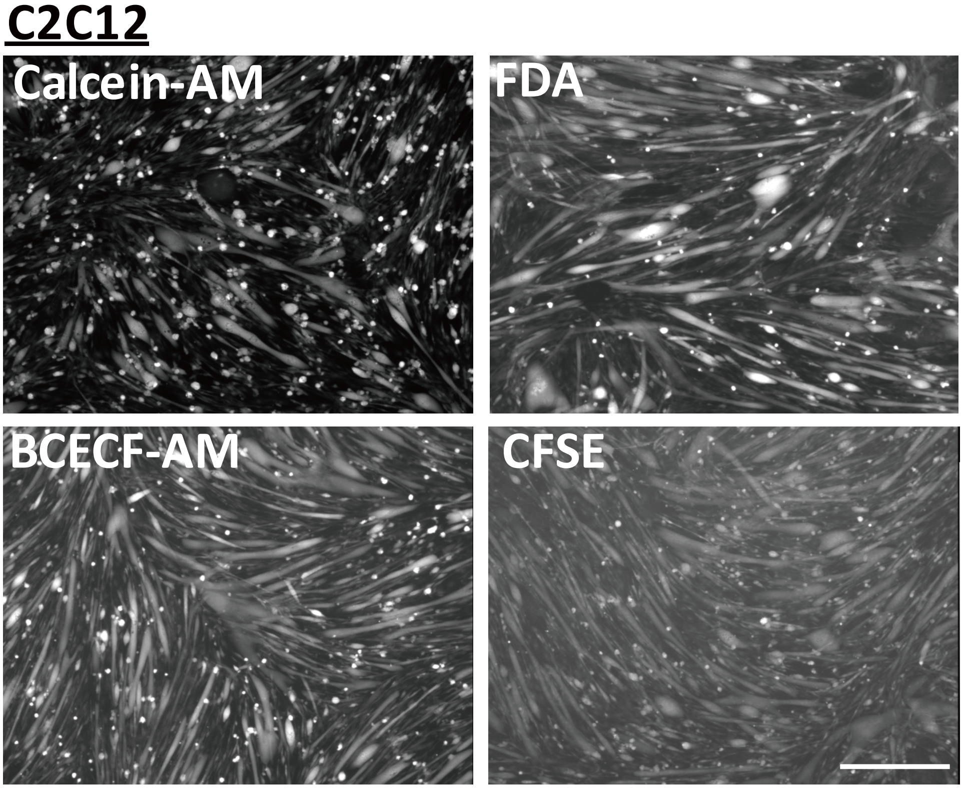

Supplementary Figure 1 | Stronger fluorescence intensity of AM-based chemicals in differentiated myotubes compared with undifferentiated myoblasts.
Representative fluorescence image of Calcein-AM-, FDA-, BCECF-AM- or CFSE-treated C2C12 myotubes 4 days after induction of differentiation. *n* = 3 for each groups. Scale bar: 500 μm.

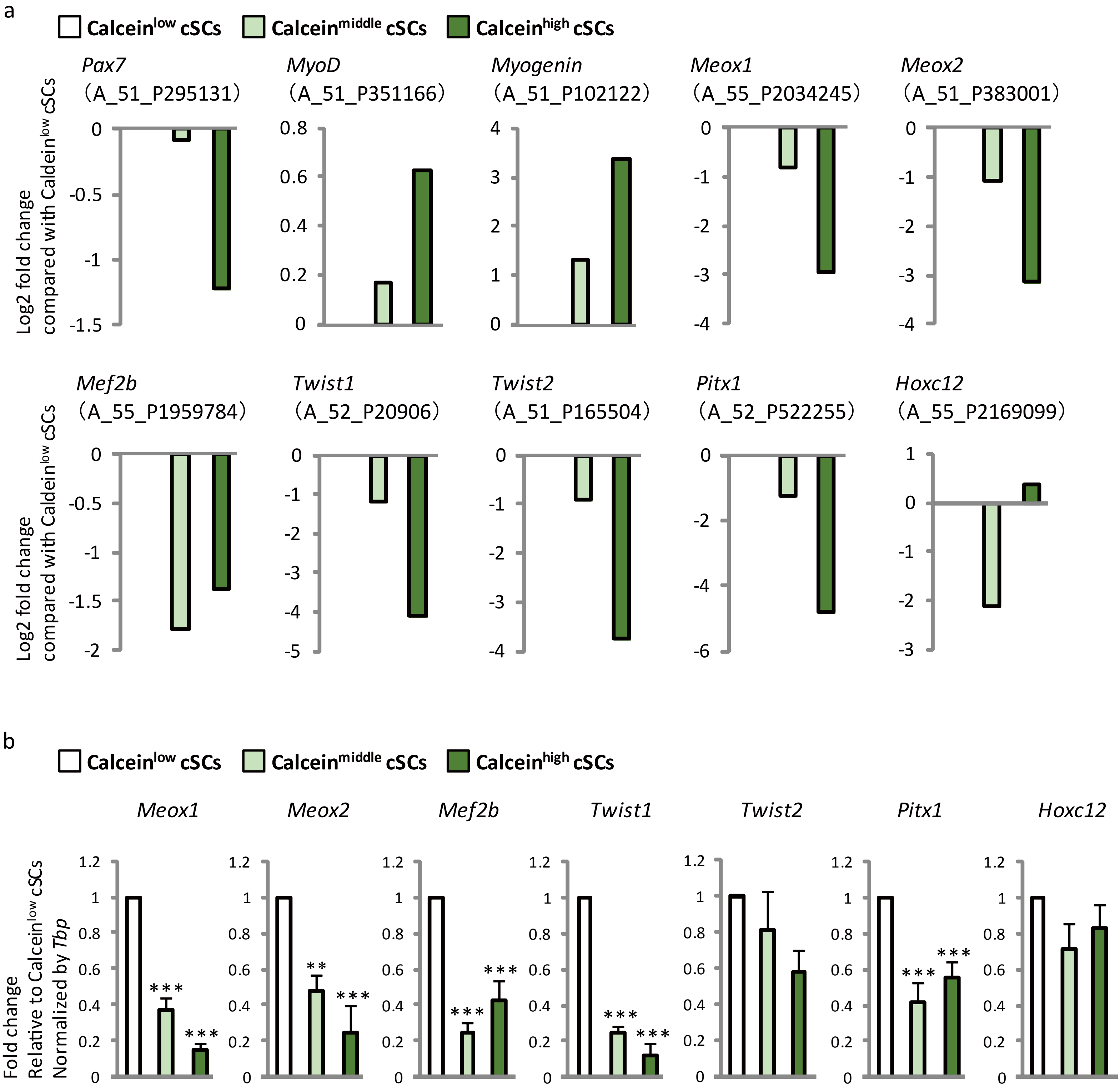

Supplementary Figure 2 | Calcein^{low} cSCs-enriched transcription factors.
Expression levels of Calcein^{low} cSCs-enriched TFs analyzed by (a) genome-wide gene expression analysis, and (b) qRT-PCR. n = 5–11. Expression of *Pax7*, *MyoD*, and *Myogenin* shown as supplement in (a). Error bars indicate SEM. *P < 0.05, **P < 0.01, ***P < 0.001 by ANOVA with Tukey–Kramer test.

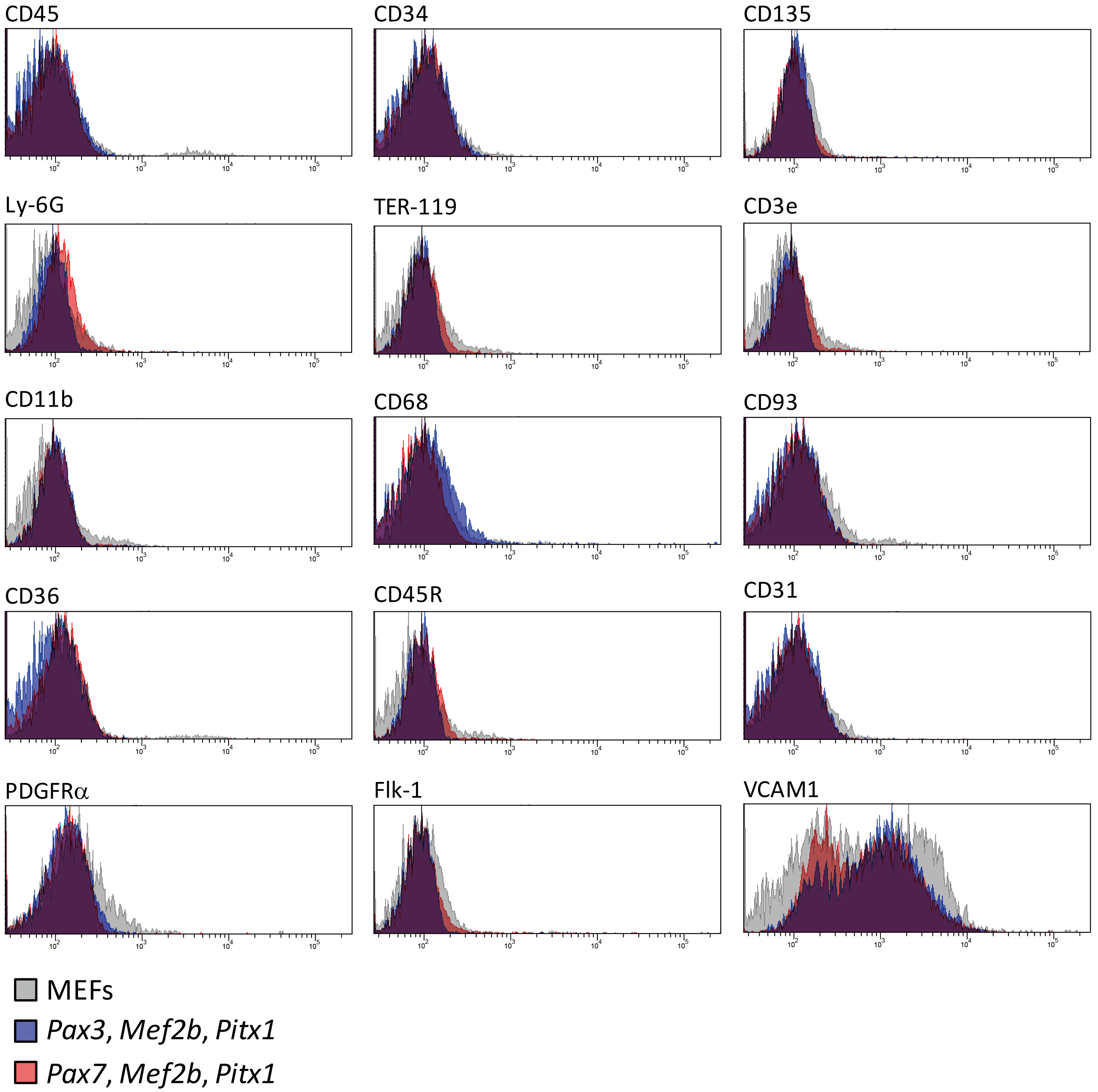

Supplementary Figure 3 | Surface marker analysis in iSkM progenitor cells. Representative FACS analysis of CD45, CD34, CD135, Ly-6G, TER-119, CD3e, CD11b, CD68, CD93, CD36, CD45R, CD31, PDGFRα, Flk-1, and VCAM1 expression in MEFs (gray). iSkM progenitor cells generated by exogenous expression of *Pax3*, *Mef2b*, and *Pitx1* (blue) or by *Pax7*, *Mef2b*, and *Pitx1* (red). *n* = 3 for each group.

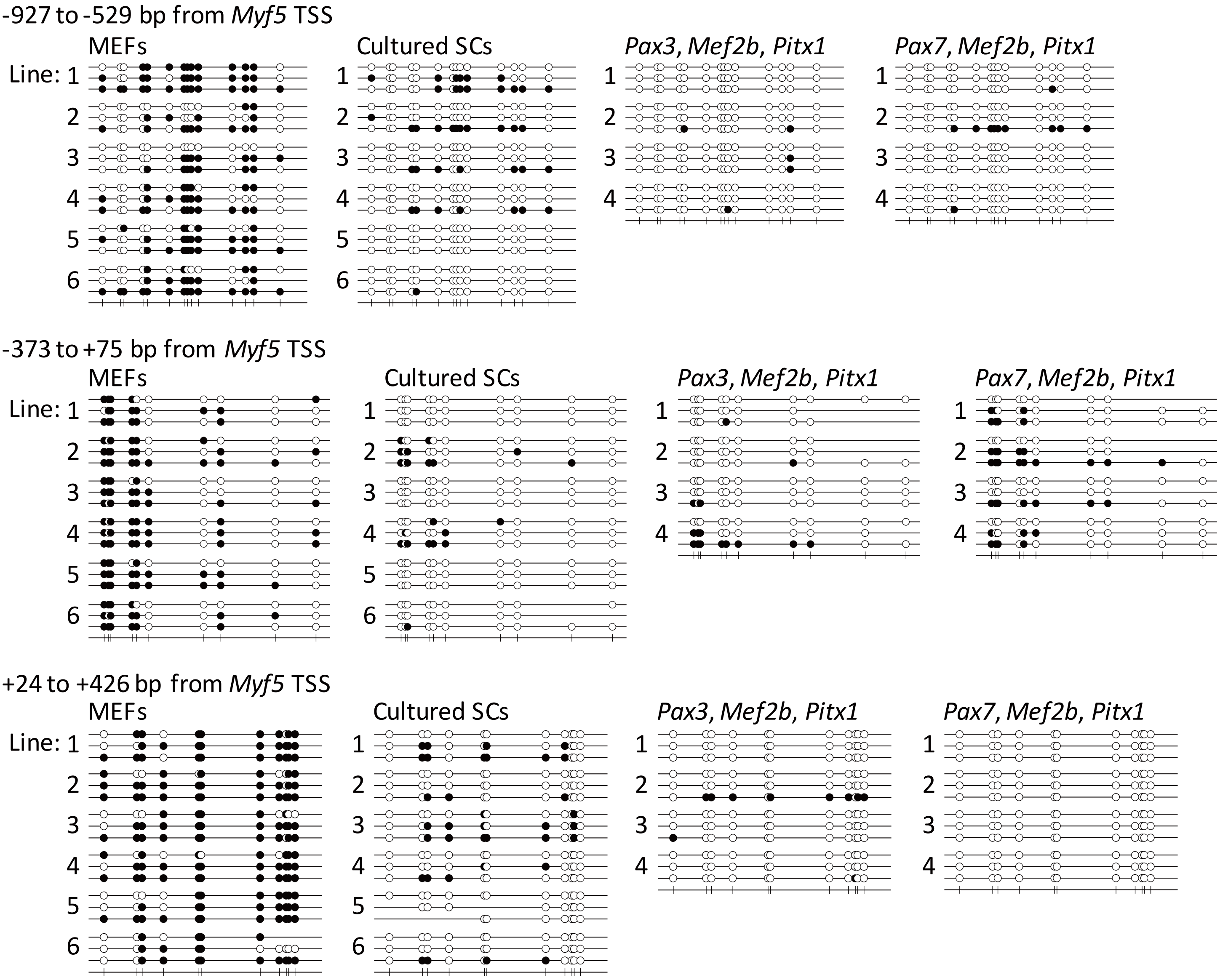

Supplementary Figure 4 | Bisulfite genomic sequencing of promoter regions of Myf5 in iSkM progenitor cells.

Bisulfite genomic sequencing of promoter regions of *Myf5* −927 to −529, −373 to +75, and +24 to +426 bp from the transcription start site (TSS) in MEFs, cultured SCs, and iSkM progenitor cells. Three obtained sequences in each line are shown. Open and closed circles indicate unmethylated and methylated CpG dinucleotides, respectively.

White adipocyte differentiation

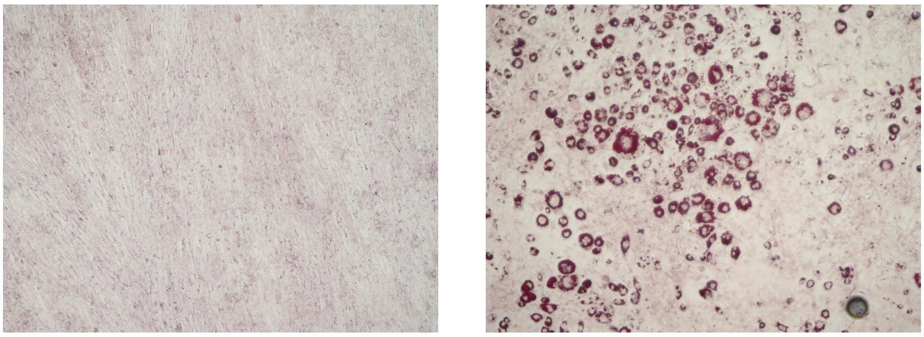

Brown adipocyte differentiation

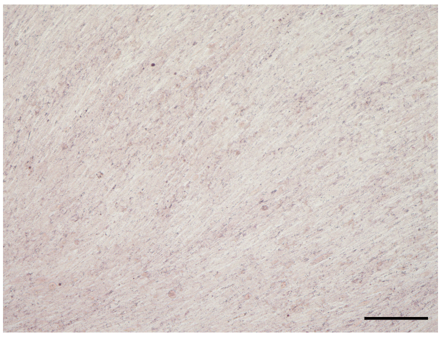

Supplementary Figure 5 | iSkM progenitor cells did not differentiate into white or brown adipocytes.

Representative Oil Red O staining for white (upper left) and brown (lower) adipogenic differentiation of iSkM progenitor cells. *n* > 4. Adipogenic differentiation of 3T3-L1 cells (upper right) is shown as a control. Scale bar: 200 µm.

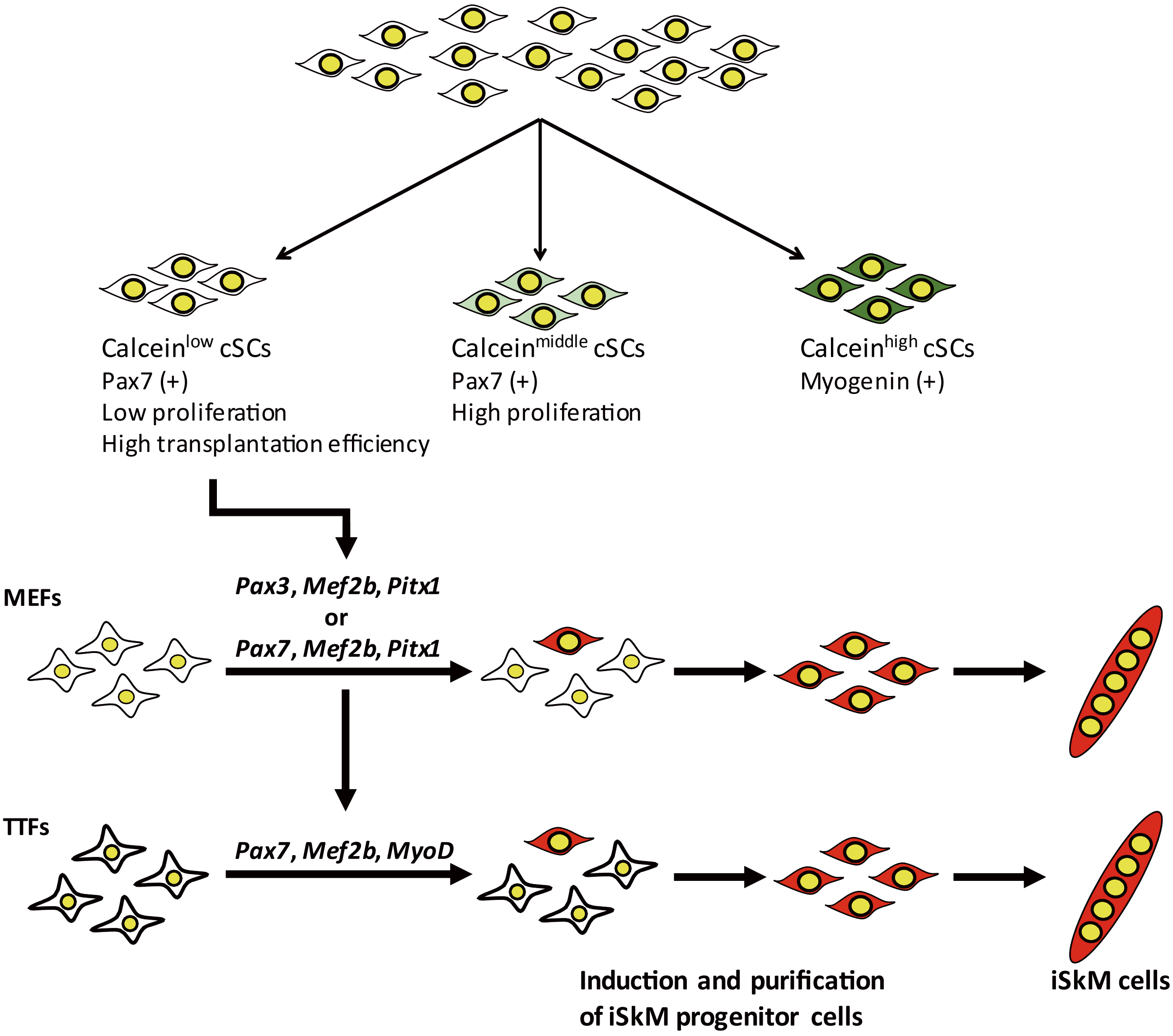

Supplementary Figure 6 | Summary of this study.
Functionally distinct subpopulations of cultured SCs were separated based on their esterase activity. Exogenous expression of Calcein^{low} cSCs-enriched TFs with *Pax3* or *Pax7* resulted in induction of iSkM progenitor cells from embryonic fibroblasts. *MyoD* was required to induce iSkM progenitor cells from adult fibroblasts.

Fig. 2b

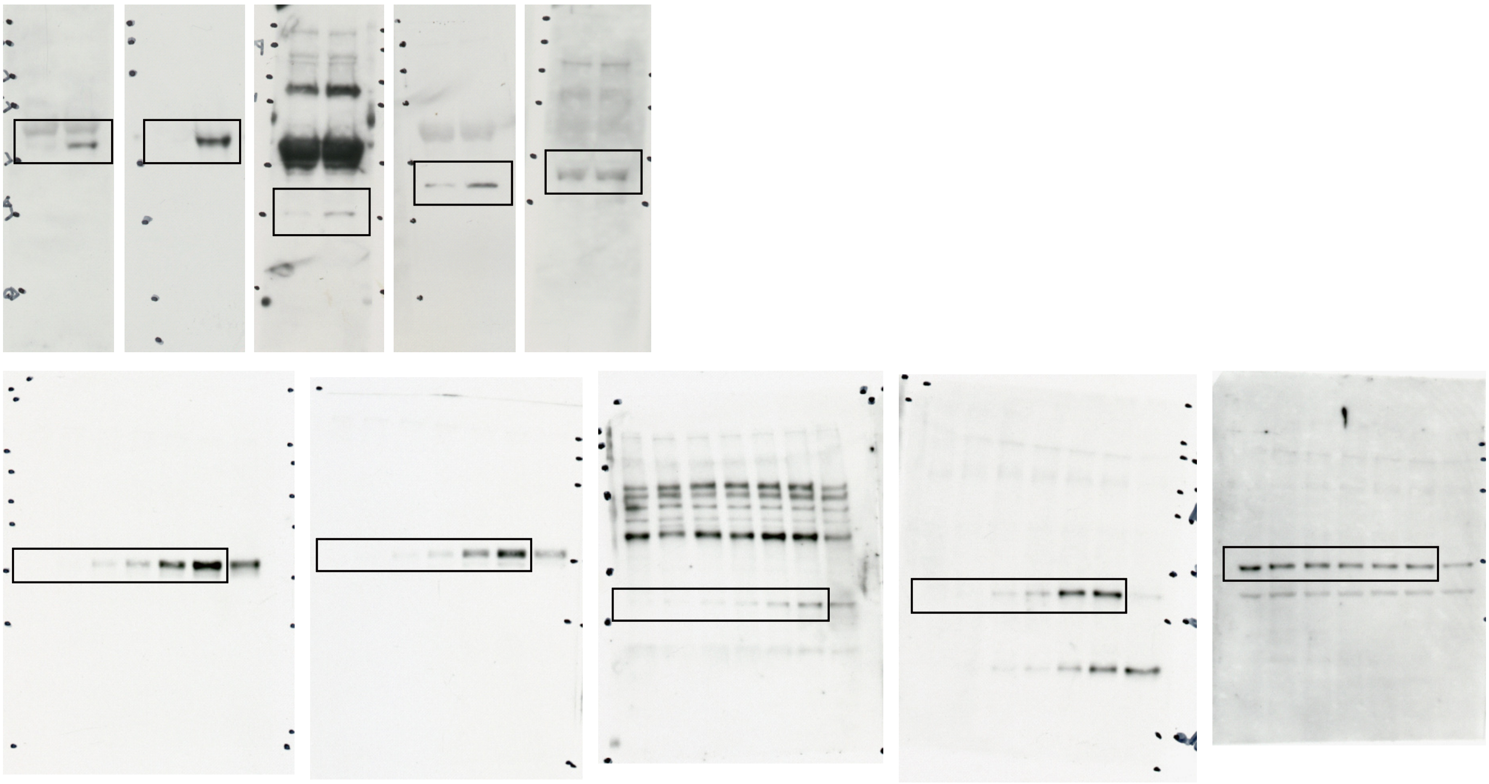

Fig. 3d

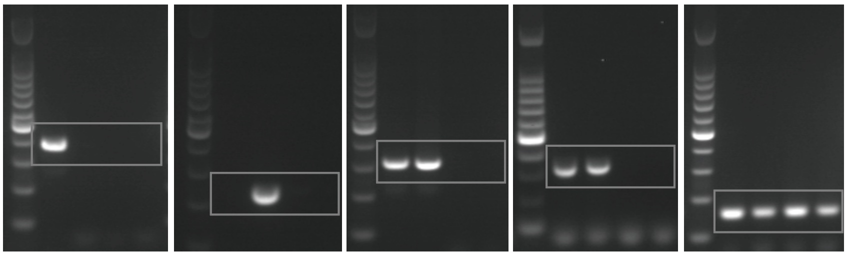

Fig. 4b

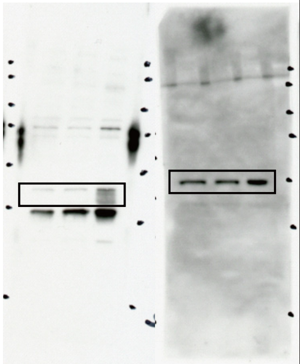

Fig. 4f

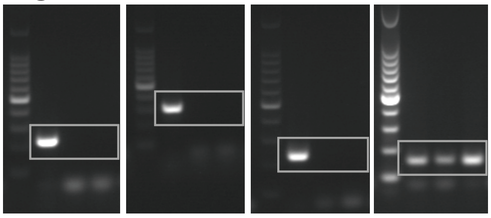

Supplementary Figure 7 | Uncropped gel data.

Genes in Calcein^{low} cSCs < Calcein^{high} cSCs
(threshold rate: 2 fold, total 2653 genes, 311 ontology)

GO Term	p-value	GO Term	p-value
myofibril	1.93E-42	muscle structure development	2.44E-20
contractile fiber	5.12E-41	developmental process	3.36E-20
sarcomere	4.77E-37	cell periphery	4.24E-19
contractile fiber part	1.58E-35	cytoskeletal protein binding	9.35E-19
I band	9.25E-25	muscle organ development	9.52E-19
muscle system process	7.90E-21	multicellular organismal development	1.72E-18
muscle contraction	9.63E-21	Z disc	2.76E-18

Genes in Calcein^{low} cSCs < Calcein^{middle} cSCs
(threshold rate: 2 fold, total 812 genes, 77 ontology)

GO Term	p-value	GO Term	p-value
contractile fiber	6.42E-20	striated muscle thin filament	5.28E-09
myofibril	4.38E-19	myosin filament	1.03E-08
sarcomere	2.03E-18	striated muscle contraction	1.70E-08
contractile fiber part	4.16E-17	myosin complex	2.04E-08
muscle contraction	2.91E-13	Z disc	2.56E-08
muscle system process	1.01E-12	actin cytoskeleton	4.24E-08
I band	3.66E-10	skeletal myofibril assembly	1.87E-07

Supplementary Table 2 | Gene ontology analysis of genes enriched in Calcein^{middle} and Calcein^{high} cSCs compared with Calcein^{low} cSCs.

Combination of TFs					Number of colony	Number of cells/colony
MyoD	*Pax3*	*Pax7*	*Mef2B*	*Pitx1*	10.75±1.08	103.5±5.02
	Pax3	*Pax7*	*Mef2B*	*Pitx1*	9.75±0.74	98.2±5.82
	Pax3		*Mef2B*	*Pitx1*	4.0±0.50	63.1±8.85
		Pax7	*Mef2B*	*Pitx1*	10.25±1.08	92.9±5.81

Supplementary Table 3 | Average number of colony and average number of cells per colony.
